# Impact Crater Hydrothermal Systems Support Rapid Microbial Enrichment and Diverse Metabolic Potential

**DOI:** 10.1111/gbi.70070

**Published:** 2026-10-17

**Authors:** Sabrina M. C. Hardy, Emily J. Aguilar‐Pine, Sean Gulick, Alaa Ahmad, Brett J. Baker, Nathalie Scott, Georgina Aitolo, Naomi Felton, Charles S. Cockell

**Affiliations:** ^1^ UK Centre for Astrobiology The University of Edinburgh Edinburgh UK; ^2^ Department of Integrative Biology University of Texas at Austin Austin Texas USA; ^3^ Institute for Geophysics & Department of Earth and Planetary Sciences, Jackson School of Geosciences University of Texas at Austin Austin Texas USA; ^4^ Center for Planetary Systems Habitability University of Texas at Austin Austin Texas USA; ^5^ Department of Marine Science, Marine Science Institute University of Texas at Austin Austin Texas USA

## Abstract

Impact crater hydrothermal systems, formed after asteroid and comet impacts, have been proposed as promising habitats for life on early Earth and as key targets in the search for life on planetary bodies such as Mars, since they provide liquid water and active geochemical turnover. Because no newly formed impact structures exist on Earth, studies of microbial establishment in impact‐crater hydrothermal systems have largely relied on the direct observation of fossil microbial life or its isotopic signatures. To address this limitation, we established simulated microcosm hydrothermal systems in the laboratory using melt‐bearing impact breccia (suevite) from the Ries impact structure, Germany, and temperate soils. Within weeks, these systems hosted taxonomically diverse communities, with broad metabolic potential, partitioned among oxidized impact glass, endolithic suevite habitats, and sedimentary environments. Dominant taxa included thermophiles from primarily heterotrophic lineages (e.g., *Meiothermus* and *Aneurinibacillus* spp.), as well as lineages with potentially chemolithotrophic metabolisms (e.g., *Brockia* spp.). Metagenomic annotations identified genes associated with methane, iron, and sulfur cycling, including sulfate‐reduction, indicating metabolic potential for biogeochemically relevant processes under hydrothermal conditions. These results show that impact‐generated hydrothermal systems can rapidly support diverse thermophilic microbial communities with habitat‐specific community structure and metabolic potential, supporting their relevance as analogue environments for studying early microbial establishment and biosignature formation in planetary impact settings.

## 1 Introduction

Impact events are a universal phenomenon, capable of transferring immense energy at the point of contact, with temperatures up to ∼15,000 K and pressures sometimes exceeding hundreds of GPa (Melosh 1989). Exposure to these extreme conditions is expected to sterilize affected substrates. This effect is largely confined to the immediate impact zone as temperatures and pressures decrease rapidly with distance from the point of impact. Like other catastrophic events that leave habitats barren, such as volcanic eruptions or glacial scouring, primary succession of organisms would be expected to occur in impact craters and their surroundings as ecosystems gradually become re‐established (Cockell and Lee 2002).

Despite the initially destructive effects, several beneficial impact‐induced alterations are known to promote microbial colonization. These can include increased permeability and porosity of target rock (Christeson et al. 2018; Cockell et al. 2002; Le Ber et al. 2022; McCall et al. 2024; Pontefract et al. 2012, 2014; Singleton et al. 2011). Immediately following impact, the thermal excursion can cause the formation of a hydrothermal system, in which fluid movement and circulation can potentially generate conditions favorable for thermophilic life (Abramov and Kring 2004, 2007; Cockell 2006; Osinski et al. 2013, 2020; Versh et al. 2006). Impact hydrothermal systems have previously been suggested as refugia for thermophilic life on the heavily‐bombarded early Earth (Abramov and Mojzsis 2009; Sleep and Zahnle 1998; although see Grimm and Marchi 2018) and as a favorable location for habitable conditions, and potentially life, on planets such as Mars (Abramov and Kring 2005; Hode et al. 2003; Newsom 1980; Osinski et al. 2013; Rathbun and Squyres 2002), where the hydrothermal system can provide sustained liquid water in an otherwise frigid planetary environment.

The duration of the hydrothermal system depends on the size of the impactor and the energy released (Arp et al. 2013). For craters with a diameter of approximately 1 km, a hydrothermal system may endure for thousands of years (Jõeleht et al. 2005; Osinski et al. 2001, 2005; Osinski 2005; Versh et al. 2005), but for craters on the order of 200 km diameter, such as the Chicxulub impact structure, the estimated lifetime may be millions of years (Kring et al. 2020).

However, owing to the lack of recent large‐scale impacts on Earth, no active surface impact‐induced hydrothermal systems currently exist, and the early biological and ecological processes in these environments remain largely speculative. While studies of present‐day impact structures have shown the presence of diverse biota, these ecosystems represent the present‐day inhabitants colonizing the post‐impact environment long after impact and after the main phase of hydrothermal activity. There have been numerous reports on fossil life associated with impact hydrothermal systems (Drake et al. 2021; Glamoclija et al. 2007; Gustafsson et al. 2025; Hode et al. 2008; Ivarsson et al. 2013; Izawa et al. 2011; Lindgren et al. 2010). Sulfur isotope excursions within post‐impact rocks have been suggested to be evidence for microbial sulfate‐reduction in post‐impact hydrothermal systems (Kring et al. 2021; Parnell et al. 2010; Simpson et al. 2017; although see Tino et al. 2023). Although these investigations show that putative fossil life is likely contemporaneous with the cooling of the impact hydrothermal system below the upper temperature limit for life, we do not know how quickly microbial communities can become established once temperatures in the hydrothermal system drop to those permissive for microbial growth, or what metabolic potential they may possess for biogeochemically relevant processes.

To address the limitations of the rock record, we assembled an artificial post‐impact hydrothermal system in the laboratory. This experiment allowed us to simulate selective aspects of the post‐impact environment and examine geological and ecological factors associated with microbial establishment, enrichment, and habitat differentiation in hydrothermally active impact crater habitats.

## 2 Materials and Methods

### 2.1 Impact Hydrothermal System Formation

To simulate impact hydrothermal systems, approximately 8.6 kg of melt‐bearing polymict breccia (termed suevite) was sampled from Aumühle quarry (48°58′17.4″ N, 10°37′49.4″ E), Ries impact structure, Germany, (described previously by Newsom et al. 1986; Osinski 2005). This material comprised a highly vesicular groundmass containing large quantities of amorphous glass as well as diverse lithic and mineral clasts. The sample was taken near, but not within, a hydrothermal pipe structure identifiable by visible discoloration (Figure 1) to preferentially select relatively weakly altered material. Nevertheless, given the age of the impact structure (approximately 14.8 million years; Buchner et al. 2013; Schmieder et al. 2018) and its documented hydrothermal history, some degree of post‐impact alteration is unavoidable. As Aumühle is an active quarry site, the material was freshly exposed and therefore at least less affected by current surface weathering.

**FIGURE 1:**
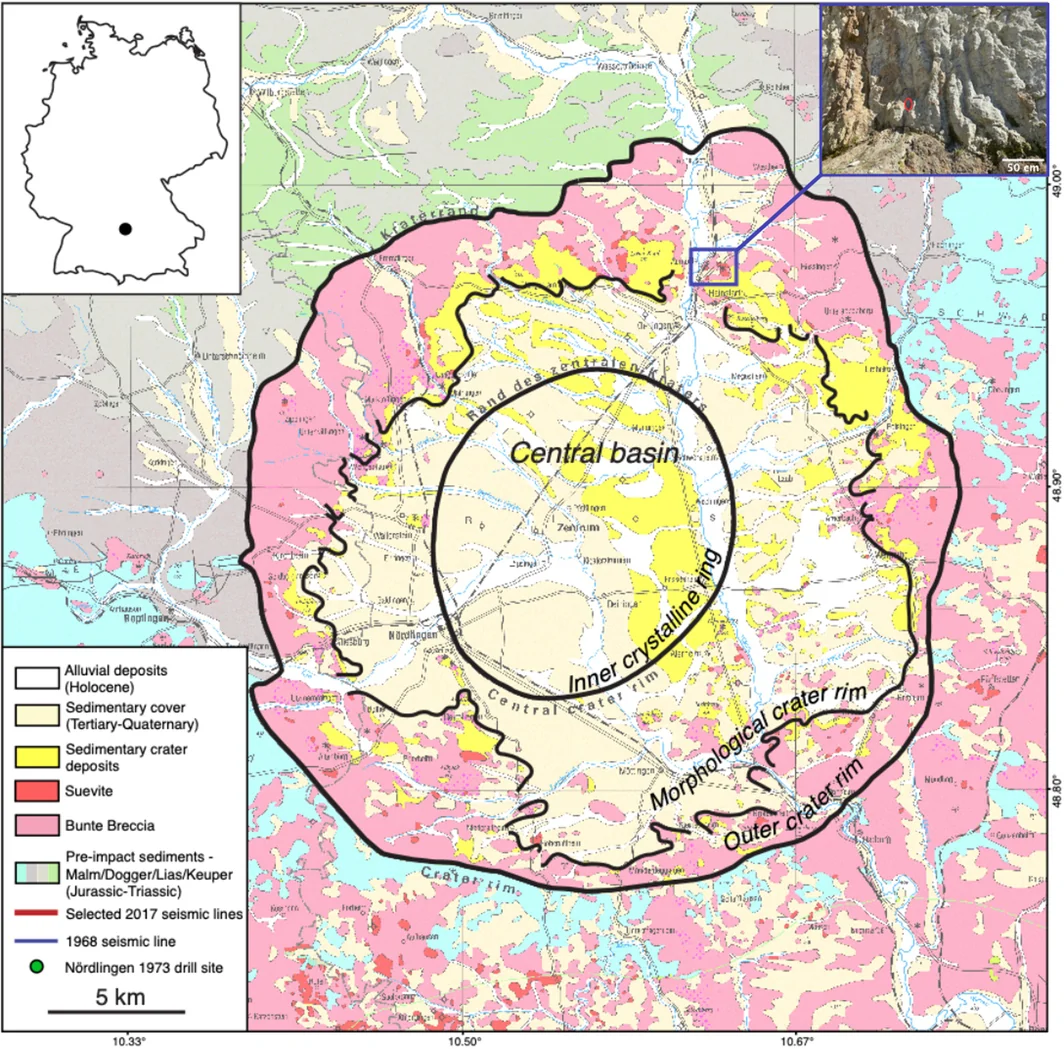
Location of Ries and source rocks. Ries Crater geologic map with location of the Aumühle suevite quarry highlighted (blue) within the ~15 Ma, 25 km diameter Ries impact structure (modified from McCall et al. 2024). Inset shows location within Germany. Upper right panel shows the sampling location within the suevite deposit in Aumühle Quarry. Hydrothermal pipes are recognizable by the discoloration; samples for this experiment were collected near but not within the pipe structures (red circle). Gray elongate clasts (“Flädle”) of impact melt are present within the suevite.

Three 750 g subsamples of suevite were randomly selected from the collected material and broken down using a sterilized rock hammer to generate multiple suevite fragments per experimental system. Impact hydrothermal systems were simulated by placing each set of rock substrate in separate water baths poised at 50°C, 60°C, and 70°C. The material in each heat bath was submerged in 1.4 L of distilled water to minimize ion input from the initial fluid and to encourage the geochemical development of fluids influenced primarily by the suevite. Each temperature condition was represented by one heat bath containing multiple suevite fragments and sampled habitats. Thus, individual rock fragments and habitat samples represent within‐system subsamples, whereas the heat bath represents the experimental unit for each temperature condition. Because the temperature systems were not replicated at the heat‐bath level, we do not treat differences among the 50°C, 60°C, and 70°C systems as statistically replicated temperature effects. Each temperature heat bath was individually covered with a sealed lid to minimize evaporation as well as contamination from the environment or between heat baths.

The three chosen temperatures lie within the range of microbial thermophily and allowed us to explore how hydrothermal temperature, (e.g., during cooling of the impact hydrothermal system or in different regions of the system) would affect microbial community composition and biogeochemical functions. At such later, lower‐temperature stages of hydrothermal evolution, impactites would already be expected to have experienced some degree of aqueous alteration. In this context, the relatively weakly altered suevite from the Ries crater provides a suitable and tractable terrestrial analogue for a substrate found in a post‐impact hydrothermal system which has cooled beneath the upper temperature limit for life.

Into each temperature condition, 10 g of soil obtained from the surface of a temperate urban site on King's Building campus of the University of Edinburgh, Scotland (55°55′21.1″ N, 3°10′29.4″ W), were added. The suevite was not sterilized, allowing any microorganisms already associated with the suevite, as well as microorganisms introduced with the soil inoculum, to be exposed to the artificial hydrothermal system. This inoculum strategy was chosen because impact structures will not necessarily be connected with existing geothermal systems, and an impact on land could experience the initial drift of soil and airborne microorganisms into the impact cavity community. We also wished to avoid the step of autoclaving suevite samples which might have altered their mineralogy. Because both unsterilized suevite and soil were included, the source of individual taxa cannot be assigned unambiguously. We therefore interpret the resulting communities as taxa that became established or enriched under the imposed hydrothermal conditions.

Furthermore, temperate soils are known to contain thermophilic microorganisms (Portillo et al. 2012). While modern crater biota has been studied as an analogue for impact‐crater‐associated ecosystems (Cockell et al. 2001, 2003, 2021; Cockell and Lee 2002), present‐day microbial communities should not be assumed to directly represent the communities that inhabited these environments immediately following impact. Rather, this experiment tests whether microorganisms drawn from plausible source environments can survive and become established under elevated post‐impact hydrothermal temperatures. Moreover, because the interior of impact craters is unlikely to be completely sterilized during the impact, surviving microbial communities could persist and interact with incoming microorganisms, contributing to the microbial source pool available for post‐impact community development.

After 12 weeks, a further 10 g of soil, sourced identically as the first sample, and 10 g of yeast extract powder (Oxoid, Thermo Fisher Scientific) were added to each heat bath for an additional 15 week‐long perturbation phase. Yeast extract was used to simulate an input of organic material, for example from necromass within or near the crater, while the additional soil introduced a second microbial input intended to test whether the established communities were stable to renewed immigration from the original source environment. Because these additions were made simultaneously, post‐week‐12 changes cannot be attributed uniquely to temporal succession, immigration, or organic enrichment. We therefore limit the interpretation of this phase to whether established microcosm communities change following transient environmental inputs.

### 2.2 Geological and Geochemical Analyzes

Suevite surface area was determined by means of the Brunauer–Emmett–Teller (BET) method via nitrogen absorption, and pore size distribution was determined via mercury intrusion analysis (both by MCA services, Cambridge).

Two thin sections were prepared and imaged using a Leica EZ4 W stereoscope. A resin round was prepared and carbon coated for investigation using a Carl Zeiss SIGMA HD VP Field Emission scanning electron microscope (SEM), fitted with Electron Backscatter Diffraction (EBSD) and Oxford AZtec ED X‐ray analysis systems. Another resin round, prepared from a sample taken from the 60°C heat bath, was likewise carbon coated and investigated using a Zeiss Crossbeam 550 focused ion beam‐scanning electron microscope (FIB‐SEM).

Powdered suevite samples, collected over the run of the experiment, were investigated via X‐Ray Diffraction (XRD) using a Bruker D8 Advance with Sol‐X Energy Dispersive detector fitted with a CoKα radiation source (λ = 1.788 °A). Additionally, powders of the altered/oxidized surface suevite were measured for sulfur (δ^34^S) and carbon (δ^13^C) isotopes by means of Elemental Analyzer Isotope Ratio Mass Spectrometry (EA‐IRMS), performed by Sercon Analytical (Cheshire, England, United Kingdom).

For Fourier Transform Infrared Spectroscopy (FTIR), 1 mg of dried, powdered samples and 198 mg (99 wt%) KBr (Uvasol, IR spectroscopy grade) were pressed into 13 mm internal diameter pellet disks and examined using a Bruker ALPHA II FTIR spectrometer.

The composition of hydrothermal fluids was monitored using an Agilent 7500ce Inductively Coupled Plasma Mass Spectrometer (ICP‐MS). Two milliliter fluid samples were fixed with nitric acid to a final concentration of 4%. During ICP‐MS, three different reaction modes were used for all samples: He, H_2_, and O_2_. Fluid pH levels were also recorded over time for each heat bath using a Jenway 3510 pH meter. A room‐temperature control was run in parallel to assess the effect of temperature on pH change, using the same suevite starting material, deionized water, and rock‐to‐water ratio (750 g:1.4 L) as the heat‐bath microcosms. The control was maintained at room temperature in a sterile falcon tube.

### 2.3 Analysis of Microbial Communities

Samples of suevite and sediments were prepared for fluorescence microscopy by staining with a 1 × 1000 working solution of SYBR^TM^ Gold dye (ThermoFisher Scientific), before incubation in the dark for 15 min. Cells were visualized under a Leica DM4000 B fluorescent microscope, using a ×100 magnification objective and blue light (I3 prism). Three repeats of cell enumerations were performed in 50 fields of view for each time point, within a 100 × 100 μm grid. A Zeiss LSM700 confocal microscope, with a ×63 magnification objective and blue light laser (488 nm, 10 mW), was used on stained samples to visualize biofilms on the surfaces of suevite fragments. Communities within the suevite were additionally imaged using a JEOL JSM6010PLUS/LV SEM on gold‐coated rock fragments.

Culture‐independent methods were used to investigate the composition of the microbial communities. DNA extractions were performed on the initial source materials and on experimental samples. Source‐material extractions included the collected suevite used in the heat baths, the week‐0 soil inoculum, and the second soil inoculum added at Week 12. Experimental samples were collected from each temperature system at Week 9, before perturbation, and Week 16, after the combined soil inoculum and organic‐matter input. At each time point, one sample was extracted from each of three habitats in each temperature system: the oxidized suevite surface above the water level, sediment, and the interior of submerged suevite fragments. DNA was extracted using the DNeasy PowerSoil Kit (QIAGEN, Germantown, MD, United States).

For bacterial community analysis, 16S rRNA gene libraries were prepared using primers 341F (5′‐CCTAYGGGRBGCASCAG‐3′) and 806R (5′‐GGACTACNNGGGTATCTAAT‐3′) targeting the V3–V4 region. Bacterial 16S rRNA gene amplicon sequencing was performed on all source‐material and experimental DNA extracts, as well as on ten bacterial isolates obtained from the heat baths. To detect archaeal sequences, primers Arch519F (5′‐CAGCCGCCGCGGTAA‐3′) and Arch915R (5′‐GTGCTCCCCCGCCAATTCCT‐3′) were used, targeting the V4–V5 region. For eukaryotes, 18S rDNA was analyzed using primers 1391f (5′‐GTACACACCGCCCGTC‐3′) and EukBr (5′‐TGATCCTTCTGCAGGTTCACCTAC‐3′) targeting the V9 region. Archaeal 16S rRNA and eukaryotic 18S rDNA sequencing were performed on a subset of samples, comprising the 50°C sediment, 60°C oxidized suevite surface, and 70°C oxidized suevite surface, selected based on their biomass and representation of distinct or potentially informative habitats. All amplicon libraries were sequenced by Novogene (Cambridge, England, United Kingdom) on an Illumina NovaSeq 6000 platform.

Shotgun metagenomic sequencing was performed on a subset of the same extracted DNA samples used for amplicon sequencing, comprising 50°C sediment and oxidized suevite surfaces from the 50°C, 60°C, and 70°C systems.

Culturing was also used to investigate which microbes would grow and to obtain isolates. Nutrient‐poor agar bacteriological medium and nutrient‐rich standard plate count agar medium (Thermo Fisher Scientific) were used, with inocula derived from habitats across the heat‐bath communities. A total of 13 enrichments and 100 subcultures were established at 60°C, with cultures distributed approximately equally between the two media. Plates were sealed in sterile airtight bags with trays of autoclaved H_2_O to minimize evaporation, and were replated every 3 days to prevent drying. Ten selected cultured samples (listed in Table S3) from heat‐bath material and fluids, were selected for DNA extraction using the DNeasy PowerLyzer Microbial Kit (QIAGEN, Germantown, MD, United States). Bacterial 16S rRNA gene sequencing was performed on these ten isolates using the 341F/806R primer pair described above.

### 2.4 Bioinformatics and Biostatistics of 16S rDNA Data

The obtained Illumina NovaSeq amplicon 16S rRNA reads were processed using the Quantitative Insights into Microbial Ecology 2 (QIIME2, version 2022.02) pipeline (Bolyen et al. 2019) by Novogene (Cambridge, England, United Kingdom). The raw sequencing data underwent several preprocessing steps to ensure high‐quality results. Initially, paired‐end reads were sorted by sample using their unique barcodes, and both the barcodes and primer sequences were removed. These reads were then assembled by merging overlapping reads using FLASH (version 1.2.11; Magoč and Salzberg 2011). Quality filtering was performed using fastp (version 0.23.1; Chen et al. 2018) to produce high‐quality clean tags. Chimeric sequences were identified by comparison with the reference SILVA database (Quast et al. 2012) and removed using the vsearch package (version 2.16.0; Rognes et al. 2016). The resulting effective tags were then processed to identify Amplicon Sequence Variants (ASVs) using the Divisive Amplicon Denoizing Algorithm (DADA2) module (Callahan et al. 2016) within QIIME2 software. ASVs were then annotated using QIIME2, employing a pre‐trained Naive Bayes classifier with the SILVA database (Quast et al. 2012).

Because each temperature condition was represented by one heat bath, we did not use the community data to test for fully replicated treatment effects. Instead, taxonomic patterns were evaluated using ASV/genera composition, alpha‐diversity indices, and relative‐abundance profiles among temperature systems, habitats, and sampling phases. Using the bacterial 16S rRNA gene amplicon ASV table generated in QIIME2, community diversity and richness was assessed with Chao1, Shannon and Simpson indices, also calculated in QIIME2. Using the same 16S data, taxonomic abundance tables (e.g., genus level Table S3) were used to visualize community composition in python (version 3.11.3). Matplotlib and Matplotlib‐venn packages (Hunter 2007) were used to generate venn diagrams, stacked bar charts and other taxa visualizations across the sampled temperatures, habitats, and timepoints.

Bray–Curtis dissimilarity was calculated from genus‐level relative‐abundance data using SciPy (Virtanen et al. 2020) and visualized as heatmaps using seaborn (Waskom 2021). Comparisons were made among all experimental samples and between experimental samples and the three inocula (Soil 1, Soil 2, and suevite). At ASV level, ASV overlap between experimental samples and the sequenced inocula was assessed to identify ASVs shared with each source community; ASVs not identified in any inoculum were classified as not detected.

### 2.5 Metagenomic Analysis

Shotgun metagenomic sequencing was performed on a targeted subset of the same DNA extracts used for amplicon sequencing. This subset comprised four samples selected to represent habitats with high biomass and/or strong community differentiation in the amplicon dataset: 50°C sediment and oxidized suevite surfaces from the 50°C, 60°C, and 70°C systems. The metagenomic data were therefore used to assess functional potential in selected representative habitats.

DNA library preparation was accomplished using the Quantabio sparQ DNA Library Prep Kit. Sequencing was performed with a 10B flow cell on the Illumina NovaSeq X sequencing platform at the University of Florida ICBR NextGen DNA Sequencing Core Facility (RRID:SCR_019152). All sample read lengths were 2 × 150 bp, and sample sequencing depth was 250 ± 63 million reads per sample. Prior to assembly, reads were quality assessed using FastQC (V0.12.1; https://www.bioinformatics.babraham.ac.uk/projects/fastqc/). FastP (v0.23.4; Chen et al. 2018) was used to remove adaptors and quality‐trim reads (parameters: ‐‐qualified_quality_phred 33 ‐p 5 ‐r cut_right_window_size = 5 cut_right_mean_quality = 15 ‐‐length_required 50). Single‐sample assemblies were generated with Megahit (v1.2.9; Li et al. 2015) (parameters: ‐m 0.5 ‐‐presets bulk). Contigs greater than 3Kb were retained for MAG generation.

Metagenomic reads were mapped to the assemblies using minimap2 (v2.28‐r1209; Li 2018) (parameters: ‐x sr ‐d ‐a) and sorted BAM files were made using SAMtools (v1.20; Li et al. 2009). The resulting coverage profiles were used for binning with MetaBAT2 (v2.12.1; Kang et al. 2019) (parameters: ‐‐minCVSum 0 ‐‐minCV 0.1 ‐‐seed 0221), MaxBin2 (v2.2.7; Wu et al. 2016) (parameters: ‐prob_threshold 0.9 ‐markerset 40), and CONCOCT (v1.1.0; Alneberg et al. 2014). DASTool (v1.1.7; Sieber et al. 2018) was then used on all output MAGs to determine the consensus MAG set (parameters: ‐‐score_threshold = 0.5 ‐‐search_engine diamond). Completeness and contamination of the consensus MAGs were evaluated using CheckM (v1.2.2; Parks et al. 2015), and only those with completeness ≥ 50% and ≤ 10% contamination were included in this analysis. To determine the relative abundance of the MAGs, metagenomic reads from four of the samples were mapped to each of the MAGs using CoverM (Aroney et al. 2025) with default parameters (v0.7.0; https://github.com/wwood/CoverM).

GTDB‐Tk (v2.4.0; Chaumeil et al. 2020) was used for the initial taxonomic classification of MAGs based on the reference GTDB‐Tk (v220; Parks et al. 2022) database. DRAM (v2.0.0‐beta5; Shaffer et al. 2020) was used with NEXTFLOW (v24.10.0; Di Tommaso et al. 2017) to annotate MAGs using curated databases (UniRef, RefSeq Viral, MEROPS, CAMPER, dbCAN, Kofam, Methyl, CANT‐HYD, VOGDB, FeGenie, Sulfur) to assign gene annotations, then distill annotations into functional categories.

To search for the metabolic pathway genes included in Figure 8, MAGs meeting high‐quality cutoffs from the CheckM results (≥ 50% complete and < 10% contaminated) were selected for gene annotation using KofamKOALA (v1.3.0, exec_annotation, −‐format mapper‐one‐line, KEGG release 117.0; Aramaki et al. 2020). The presence/absence of MAG gene annotations within each metagenomically analyzed sample and selected major metabolic pathways are shown in Figure 8, with specific pathway marker genes compiled in Table S8.

To identify dsrA genes, predicted protein sequences from PRODIGAL (v2.6.3; Hyatt et al. 2010) of the metagenomically analyzed assemblies were searched against the SILVA dsrA reference database (release 2024‐09‐23; Müller et al. 2015; https://www.arb‐silva.de/archive/dsrAB/) using DIAMOND (v2.1.8.162; Buchfink et al. 2015, 2021) and blastp (‐‐max‐target‐seqs 1). Sample sequences were combined with the SILVA reference sequences. MAFFT (v7.526; Katoh and Standley 2013) was used to create an alignment of the sample and reference sequences. The resulting alignment was trimmed using ClipKit (v1.3.0, −m smart‐gap; Steenwyk et al. 2020). The final dsrA protein phylogenetic tree was inferred with IQ‐TREE (v2.0.7; Minh et al. 2020) using 1000 bootstrapping replicates (‐bb 1000). The best model generated, LG + R10 was selected according to Bayesian information criteria, and the tree was visualized with iTOL (Letunic and Bork 2024). Selected sample sequences included in the dsrA protein phylogeny are compiled in Table S8.

## 3 Results

### 3.1 Geological and Geochemical Alteration

BET analysis and mercury intrusion porosimetry of the suevite provided surface area values of 28.902 m^2^/g and 30.770 m^2^/g, respectively (Figure S1). The latter also provided a porosity value of 42.11%, as well as a pore diameter distribution which identified ∼60% of pore diameters as greater than 1 μm (which we deem potentially accessible to microorganisms). This is a maximum percentage estimate as information on pore throat diameters (i.e., permeability) is not considered in the intrusion analysis. The material was made up of diverse mineral and glass clasts including iron‐rich regions (Figures S2–S4), consistent with previous geological analysis at Ries (Osinski et al. 2004). The presence of clay minerals and partially altered impact glass indicates low‐temperature aqueous alteration, consistent with the known hydrothermal history at Ries crater.

Within the first week of exposure to the simulated post‐impact hydrothermal system, the impact suevite underwent hydrothermal alteration. Layers of red alteration occurred on surfaces of rocks exposed above the water level, most conspicuously in the 70°C heat bath (Figure 2; Figures S5–S7). These changes did not occur on the exterior of submerged suevite, or in the interior of any of the rocks, suggesting that the observed surface changes were associated with atmospheric exposure.

**FIGURE 2:**
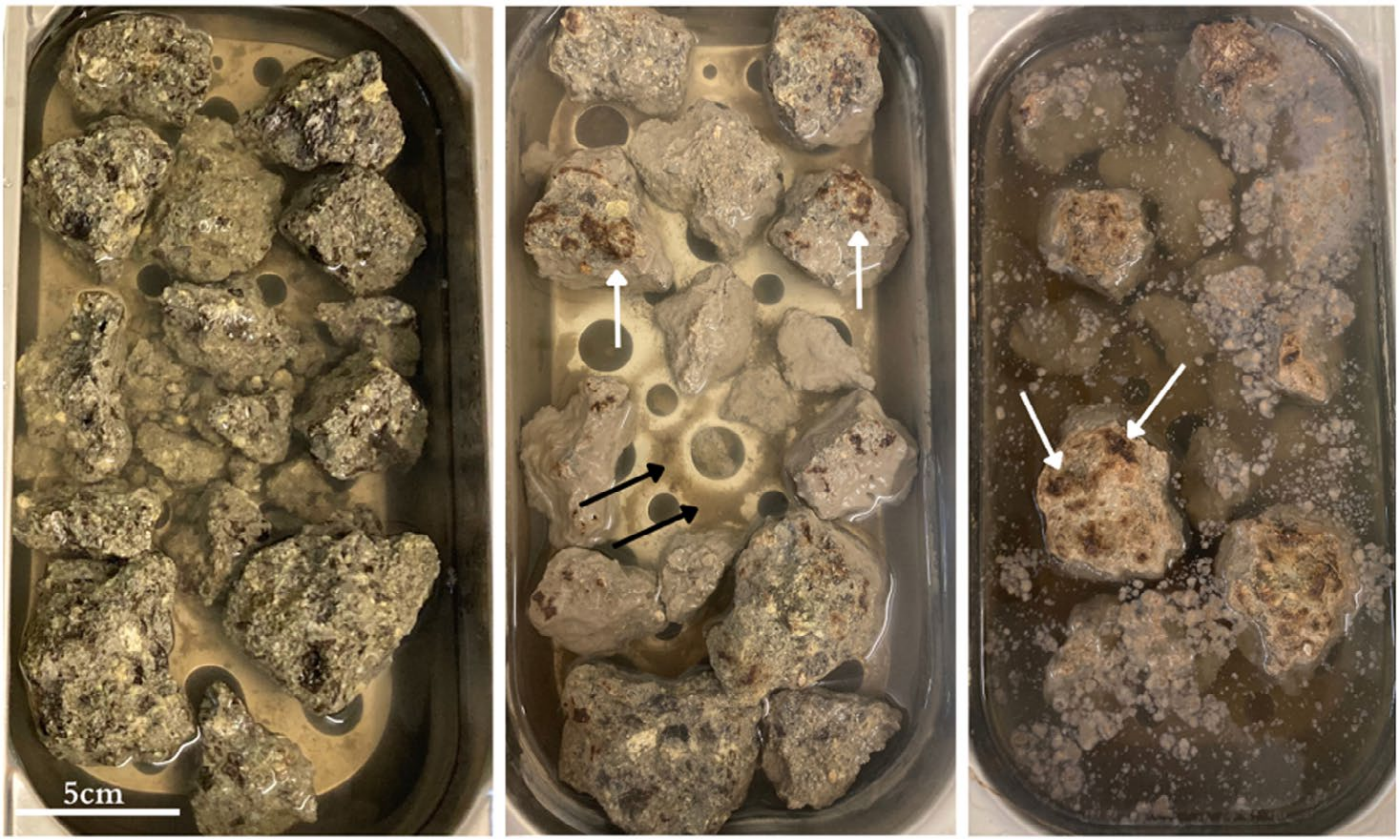
Development of the impact hydrothermal system. Alterations associated with the 70°C artificial post‐impact hydrothermal system. Images taken on week 0 (left), 6 (middle), and 13 (right). Organics and the second soil inoculum were added on Week 12. Black arrows indicate the accumulating sediment from the dissolution of suevite. Red alteration to suevite surfaces exposed to the atmosphere are indicated with white arrows, with alteration increasing over time.

According to XRD, the Ries impact suevite was mostly composed of smectites (comprising on average 21 wt% of the material) and impact glass‐bearing regions (observed as an amorphous ‘hump’ in the diffractogram and making up on average 32 wt%). Other crystalline components within the suevite were quartz (15 wt%), plagioclase (14 wt%), mica (8 wt%), K‐feldspar (4 wt%), calcite (3 wt%), and talc (3 wt%) (Figure S8; Table 1). Small quantities of apatite, zircon, kaolinite and ilmenite were additionally identified via SEM‐EDS (Figure S3). The red alteration zones were found to be similar in composition (Table S1) and were spatially associated with a calcium‐rich rind which formed on the sample surface (Figure 5). Further investigation by XRD on week 17 revealed that calcite wt% was approximately double that in the bulk suevite (5–6 wt%) in these regions (Table S1). We interpret the red colouration as consistent with incorporation of oxidized iron within the calcite, reflecting formation or alteration under oxidizing conditions in contact with the atmosphere. These observations would be consistent with previous observations of high iron‐containing glasses in the Ries suevite (Osinski et al. 2004).

**TABLE 1:** Quantification of microbial diversity in the hydrothermal system.

Sample	Temperature (°C)	Chao1	Shannon	Simpson
**Inoculants**				
Raw suevite	—	171.5	6.20	0.98
Frist soil	—	966.5	6.71	0.99
Second soil (with organics)	—	1020	6.76	0.99
**Habitats—Week 9**				
Endolithic	50	9.0	2.76	0.83
	60	88.88	4.73	0.93
	70	40.67	4.08	0.90
Oxidized surface	50	166.2	5.87	0.98
	60	129.7	4.49	0.89
	70	85.8	4.85	0.94
Sediment	50	190.5	5.67	0.97
	60	48.1	4.40	0.93
	70	42.1	3.40	0.78
**Habitats—Week 16**				
Endolithic	50	93.1	4.92	0.95
	60	114.3	4.90	0.93
	70	73.3	4.23	0.89
Oxidized surface	50	102.0	5.60	0.97
	60	122.6	5.15	0.95
	70	115.4	4.99	0.95
Sediment	50	185.1	5.57	0.97
	60	127.4	5.15	0.96
	70	77.2	3.74	0.84

*Note:* Quantification of microbial diversity in the hydrothermal system. Chao1, Shannon, and Simpson diversity indices based on 16S rRNA gene amplicon sequencing are shown for the sampled hydrothermal habitats across the 50°C, 60°C, and 70°C systems. Source‐material samples, including the suevite substrate and soil inocula, are also included for comparison.

Some of the suevite disaggregated, accumulating on the surface, around and underneath the intact suevitic rocks (black arrows, Figure S9) forming a sedimentary layer made up of material with grain sizes ranging from a few tens of microns to millimeter sizes, as well as some larger suevite fragments. Thus, we recognized the rapid formation of three distinct potential hydrothermal rock habitats: oxidized surface suevite, submerged suevite—particularly endolithic habitats within these rocks—and a sedimentary habitat made up of disaggregated suevitic material.

Geochemical changes were observed in the fluids within the system, with gradually increasing fluid alkalinity and elemental concentrations over time. A final pH of 8.97, 8.99, and 9.17 (initial pH 7.7) was achieved in the 50°C, 60°C and 70°C heat baths, respectively. A 25°C control achieved a final pH of 8.3 (Figure S9). The observed increase in pH within the heat baths is consistent with alkaline fluid development during suevite–water interaction and can be considered alongside independent nitrogen isotope evidence reported from the Ries crater (Stüeken et al. 2020). ICP‐MS data detected measurable concentrations of potentially bioavailable elements in all heat‐bath fluids (Figure 3; Figure S10). Although minor contributions from the steel trays or external dust cannot be excluded, each tray was individually covered with a sealed lid to minimize environmental contamination and cross‐contamination between trays. These geochemical data provide environmental context for interpreting microbial community development, including the availability of elements relevant to microbial metabolism, but do not directly demonstrate biological control of fluid chemistry.

**FIGURE 3:**
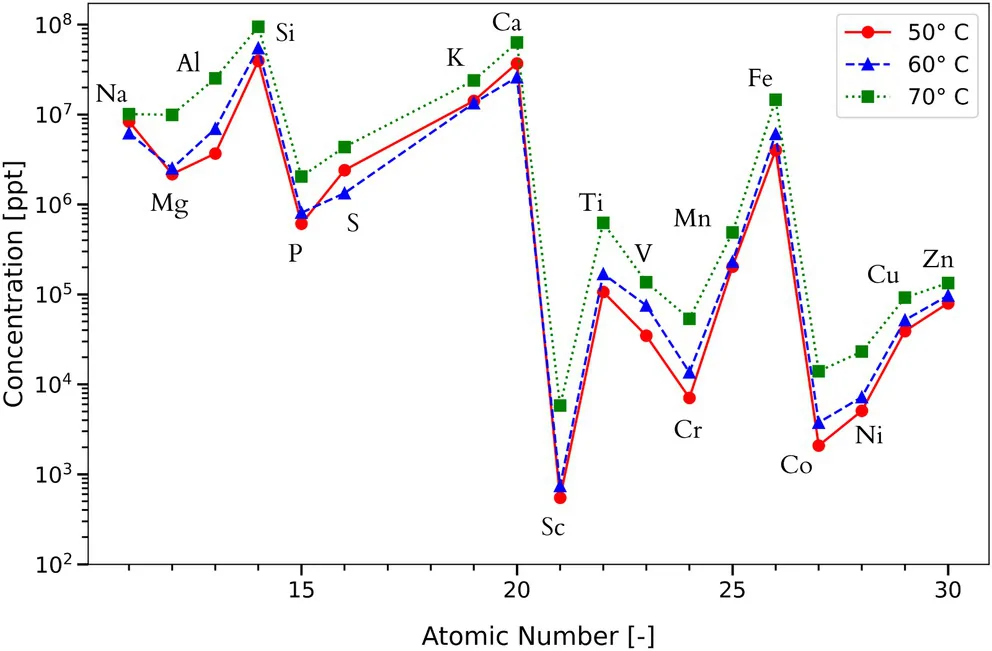
Geochemical composition of hydrothermal fluids at different temperatures. Absolute concentrations of selected major and trace elements, including elements relevant to microbial metabolism, arranged by atomic number from 11 (Na) to 30 (Zn), measured via ICP‐MS after 12 weeks in the artificial hydrothermal system at 50°C, 60°C, and 70°C.

### 3.2 Biofilm Formation on Hydrothermal Impact Substrate

Within 12 weeks of the start of hydrothermal activity, we observed the establishment of microbial communities within all three habitats. Figure 4 shows examples of microbial habitats and biofilms studied by microscopy.

**FIGURE 4:**
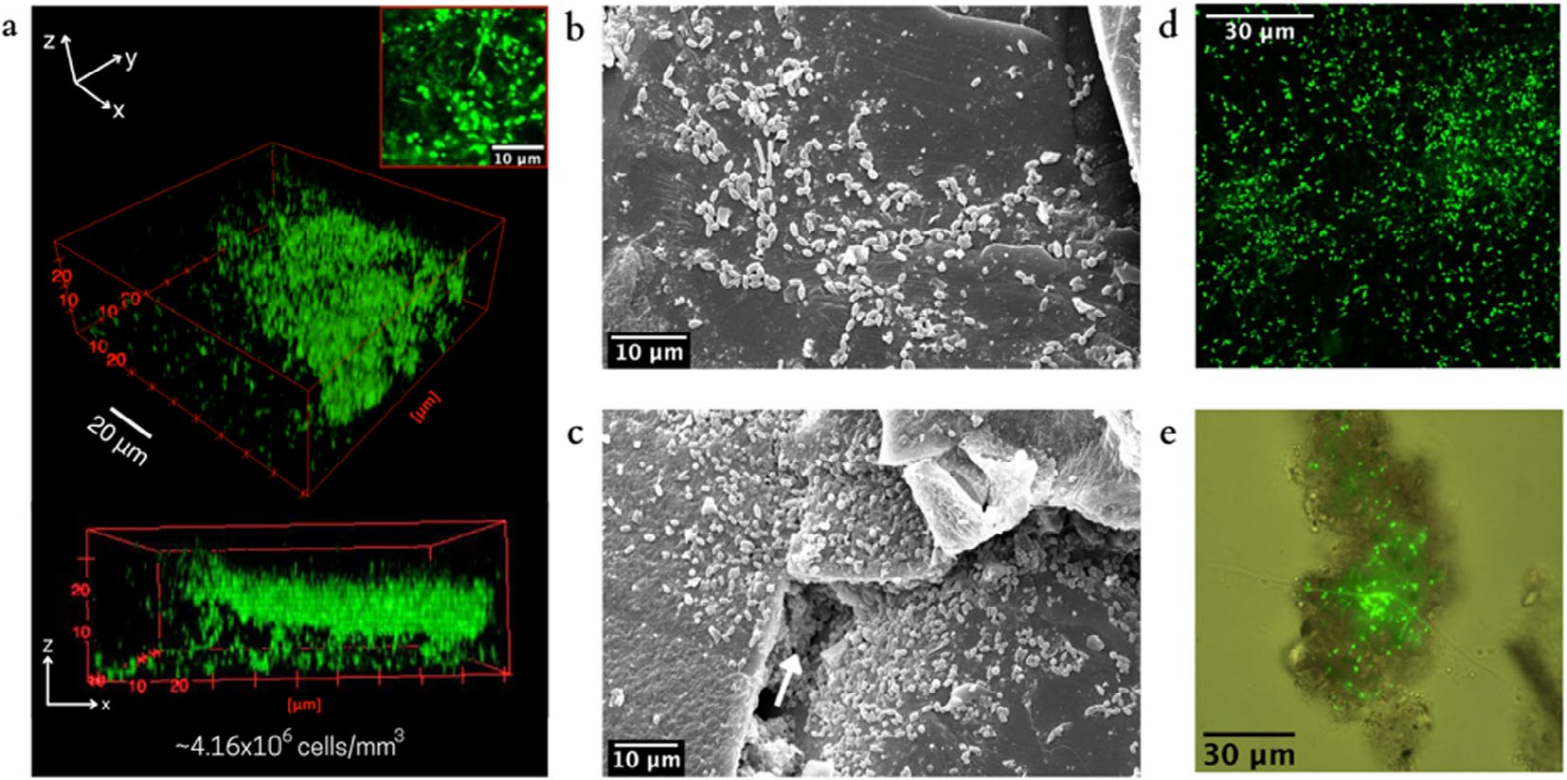
Biofilm formation and microbial habitats in artificial impact hydrothermal system. (a) Red altered surface: Confocal microscopic examination of a biofilm formation on oxidized suevitic surfaces from 60°C showing top down and side‐on views of SYBR Gold stained cells. (b, c) Interior of submerged suevite: Scanning electron microscopy of cross sections of suevite from 50°C revealing colonies of endoliths on planes (b) and along grain boundaries (c, white arrow). (d) Interior of submerged suevite: Confocal microscopic examination of a cross section of suevite from 70°C showing SYBR Gold stained endoliths. (e) Sediment grain: Fluorescence microscopy showing SYBR Gold‐stained microbes attached to the surfaces of a grain from 50°C, overlayed onto brightfield image.

Biofilm formation was observed within the oxidized suevite zones, with growth found to be densest at 60°C. These surfaces contained the largest quantities of biomass of the habitats which was detectable by Confocal microscopy and Fourier Transform Infrared Spectroscopy (FTIR) (Figures 4a and 5; Figures S11 and S12). Biofilms were spatially associated with calcium‐rich rinds, and SEM revealed pit‐like features within the calcite that were similar in size to microbial cells (Figure 5d). These observations are consistent with possible microbial interaction with mineral surfaces, but do not distinguish between biologically influenced and abiotic calcite formation. XRD showed that the highly oxidized regions were still primarily composed of impact glass (Table S1).

**FIGURE 5:**
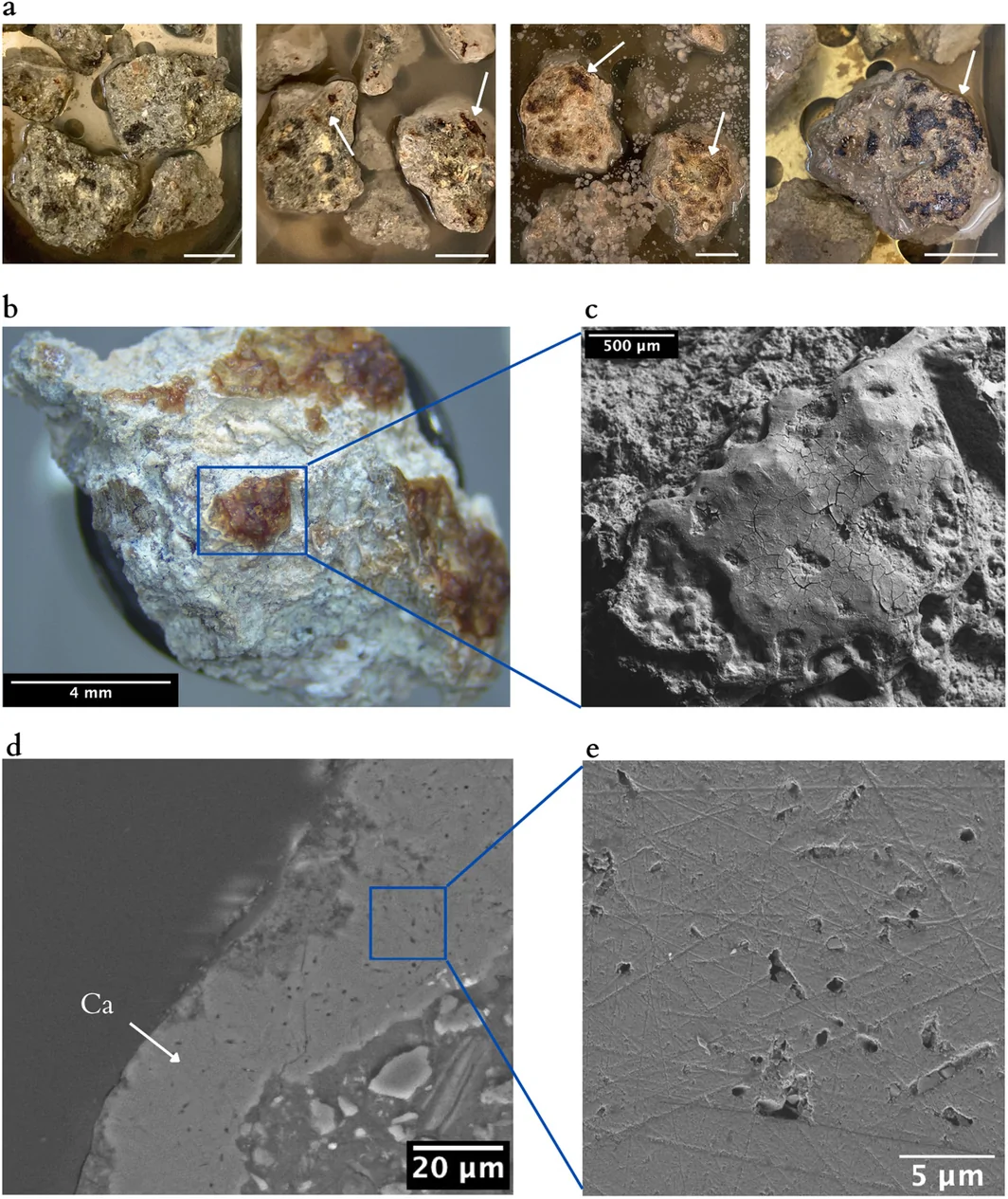
Microscopy images of oxidized suevite surfaces and carbonate formation. (a) Photographic image of the same few suevite samples in the 70°C heat bath after 1, 8, 13, and 20 weeks. White arrows indicate newly formed, darkening oxidized patches. Scale bars are 2 cm. (b) Photographic image of rock chip with newly altered surface from the 60°C heat bath on Week 13. (c) Scanning Electron Microscope (SEM) secondary image of altered surface. (d) SEM secondary image of altered suevite sample in resin round (from 60°C heat bath on Week 16) where point EDX (energy‐dispersive X‐ray spectroscopy) identified a calcium rich (Ca: Calcium carbonate) rind. (e) SEM secondary image of Ca‐rich rind with microbial size holes.

Endolithic habitats within submerged suevite hosted microbes, deeply distributed into the rock matrix (Figure 4b,c; Figure S13). They were observed along grain boundaries, on surfaces (possibly fracture surfaces or cleavage planes), and inside pore spaces, taking advantage of the impact‐induced brecciation and porosity of the material (Muttik et al. 2008). In the sedimentary habitat, microbes were observed attached to grain surfaces as either isolated cells or as part of an assemblage of microorganisms growing on the surface of the grains (Figure 4d,e; Figure S14). As XRD indicates that the sedimentary habitat has a composition similar to the original suevite, these grains likely consist of a mixture of clay minerals along with mineral grains derived from the mineral and lithic clasts present in the suevite. The sedimentary clay might also be regarded as part of a (fourth) planktonic habitat since this material often remained suspended within the fluid. In a natural impact structure, clay‐rich alteration zones derived from suevite may provide favorable sites for microbial colonization. Hydrothermal fluid flow could then redistribute cells from these colonized zones to new rock surfaces within the system.

The presence of organic material within the habitats was confirmed by Fourier Transform Infrared Spectroscopy (FTIR) (Figure S15) which showed the strongest peaks from organic carbon associated with the oxidized suevite at 60°C, consistent with confocal microscopy observations. The δ^13^C values of all oxidized surface samples were higher than that of the Reis suevite starting material (−27.27‰), indicating a modest enrichment in ^13^C of 0.60‰–1.02‰ (Table S2). Sulfur concentrations were below the analytical threshold (< 0.02%) in all samples and were therefore too low for reliable δ^34^S analysis.

### 3.3 Microbial Diversity and Metabolic Potential in Impact Hydrothermal Substrates

After 12 weeks, we observed that the simulated hydrothermal systems hosted taxonomically diverse microbial communities, with community composition varying among sampled habitats (Figure 6; Table 1; Figure S16). Culture‐independent analysis on all samples taken from the heat baths identified 30 phyla, 80 classes, 161 orders, 238 families, 340 genera, and 141 species distributed across the temperature systems and sampled habitats (Figure 6; Figures S17 and S18; Table S3).

**FIGURE 6:**
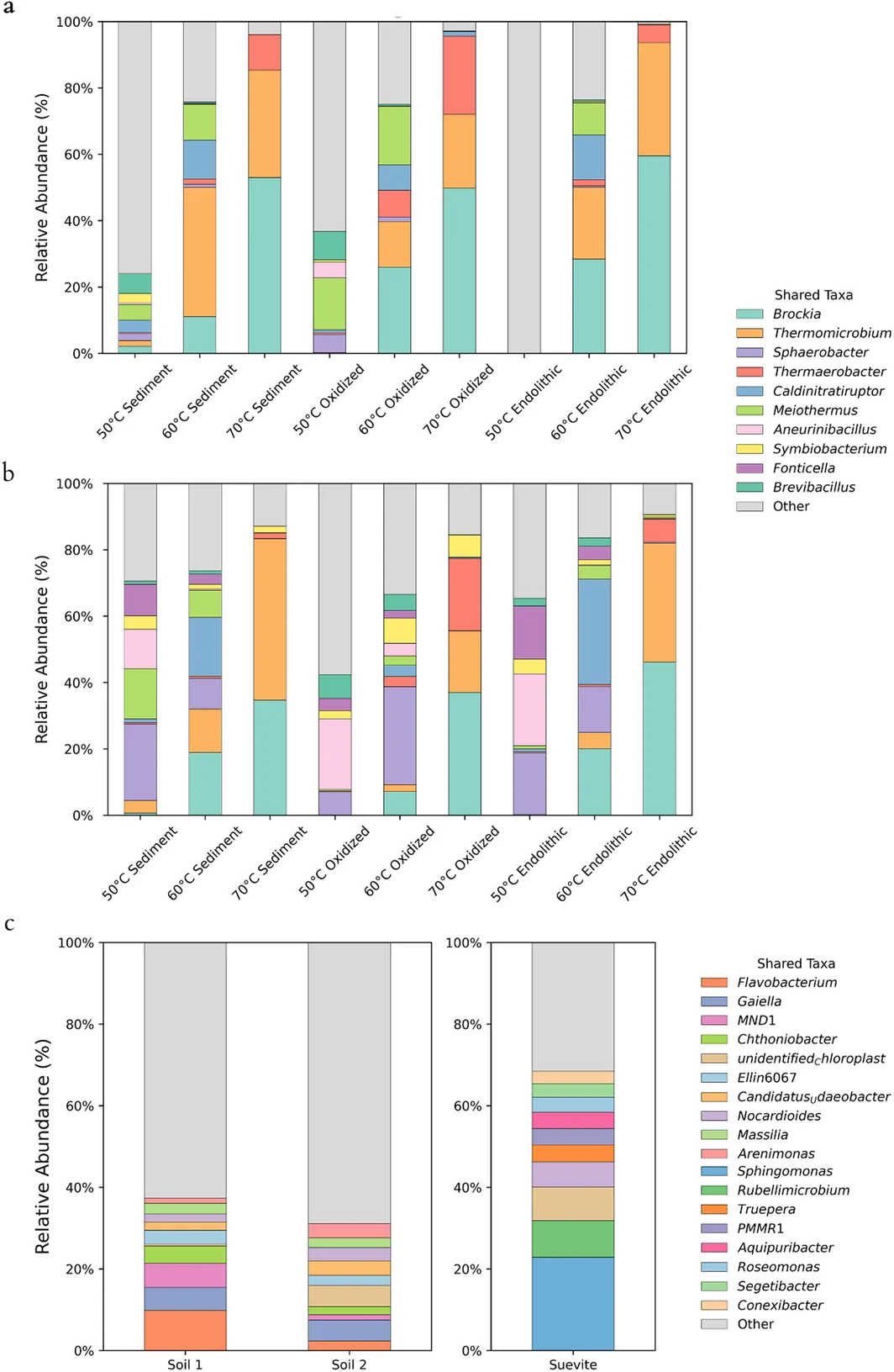
Bacterial community composition in the impact hydrothermal system. Stacked bar charts showing the relative abundance of the top 10 shared genera based on bacterial 16S rRNA gene amplicon sequencing. Samples collected after (a) 9 weeks and (b) 16 weeks from different habitats, comprising sediment, oxidized surface, and endolithic suevite, within each temperature system (50°C, 60°C, and 70°C). (c) Top 10 shared genera in source‐material samples, including the two soil inocula and the suevite substrate. Shared taxa are shown to highlight similarities in bacterial community composition across samples. Relative abundances are normalized within each sample, with all remaining taxa grouped as “Other.”

The two soil inocula showed broadly similar dominant genera, consistent with their collection from the same source environment, whereas the suevite source material showed a more distinct community composition (Figure 6). The heat‐bath communities were dominated by genera that differed from those most abundant in the source materials, indicating that the imposed hydrothermal conditions were associated with marked shifts in dominant community composition. Genus‐level Bray–Curtis comparisons supported this pattern, with all experimental communities showing high dissimilarity from the inocula (minimum distance = 0.95). Similarly, ASV comparisons showed that > 60% of ASVs in every experimental sample were not detected in any of the three inocula. This may reflect strong selection during the transition from temperate source communities to thermophilic conditions; some taxa may have been present below detection limits in the inocula but subsequently enriched under the more extreme conditions, while spore‐forming taxa may have been less effectively recovered during DNA extraction.

Dominant bacterial groups in the microcosms included thermophilic and heat‐tolerant representatives, particularly Firmicutes and Chloroflexi, with additional contributions from Proteobacteria, Actinobacteriota, Gemmatimonadota, and Acidobacteriota (Figure S18). At the genus level, the 50°C habitats showed more variable dominant community composition (50°C endolithic week 9 was the most dissimilar habitat; Figure 6; Figure S16), while the higher temperature habitats shared a more consistent set of dominant thermophilic genera (e.g., *Brockia* and *Thermomicrobium*) across habitats (Figure 6). This pattern could suggest that higher‐temperature systems were associated with a less diverse set of dominant thermophilic genera, while the lower‐temperature system supported more variable dominant community compositions among habitats.

Sequencing of ten selected cultured samples recovered thermophilic and heat‐tolerant genera, including *Thermaerobacter, Geobacillus, Brevibacillus, Thermomicrobium, Symbiobacterium*, and *Sphaerobacter* (Table S3). These cultured representatives were also detected in the culture‐independent dataset, indicating that a subset of the heat‐bath community was recoverable under the thermophilic cultivation conditions used.

Three months into our experiment, we added a further 10 g of soil inoculum and 10 g of yeast extract to each heat bath as a combined microbial‐inoculum and organic‐matter perturbation. Yeast extract was used to simulate organic input in the form of decaying biomass, while the additional soil inoculum was intended to test whether the established communities were stable to renewed immigration from the original source environment. Following this combined perturbation, cell numbers increased (Figure S19), and the number of taxa unique to these habitats changed, with some taxa becoming enriched. For example, the endolithic 70°C suevitic habitats increased from 11 genera before the perturbation to 193 genera after the perturbation (Figure S17). These shifts indicate that microbial communities in impact hydrothermal microcosms can change substantially following transient environmental and biological inputs.

We identified many taxa and genes with potential relevance to biogeochemical processes in hydrothermal systems (Tables S4 and S5). Sequences affiliated with sulfate‐reducing genera (e.g., *Desulfofundulus* and *Desulfotomaculum*) were identified and metagenomic analysis corroborated this identifying *dsr* (dissimilatory sulfite reductase) genes belonging to a diversity of taxa including *Desulfallas*, *Desulfofundulus*, *Thermodesulfobium* and *Thermanaeromonas* spp., as well as diverse members of Firmicutes. A phylogenetic analysis of dsrA protein sequences from metagenomically analyzed samples confirmed this diversity, with sequences from multiple sample conditions distributed across multiple lineages (Figure 7).

**FIGURE 7:**
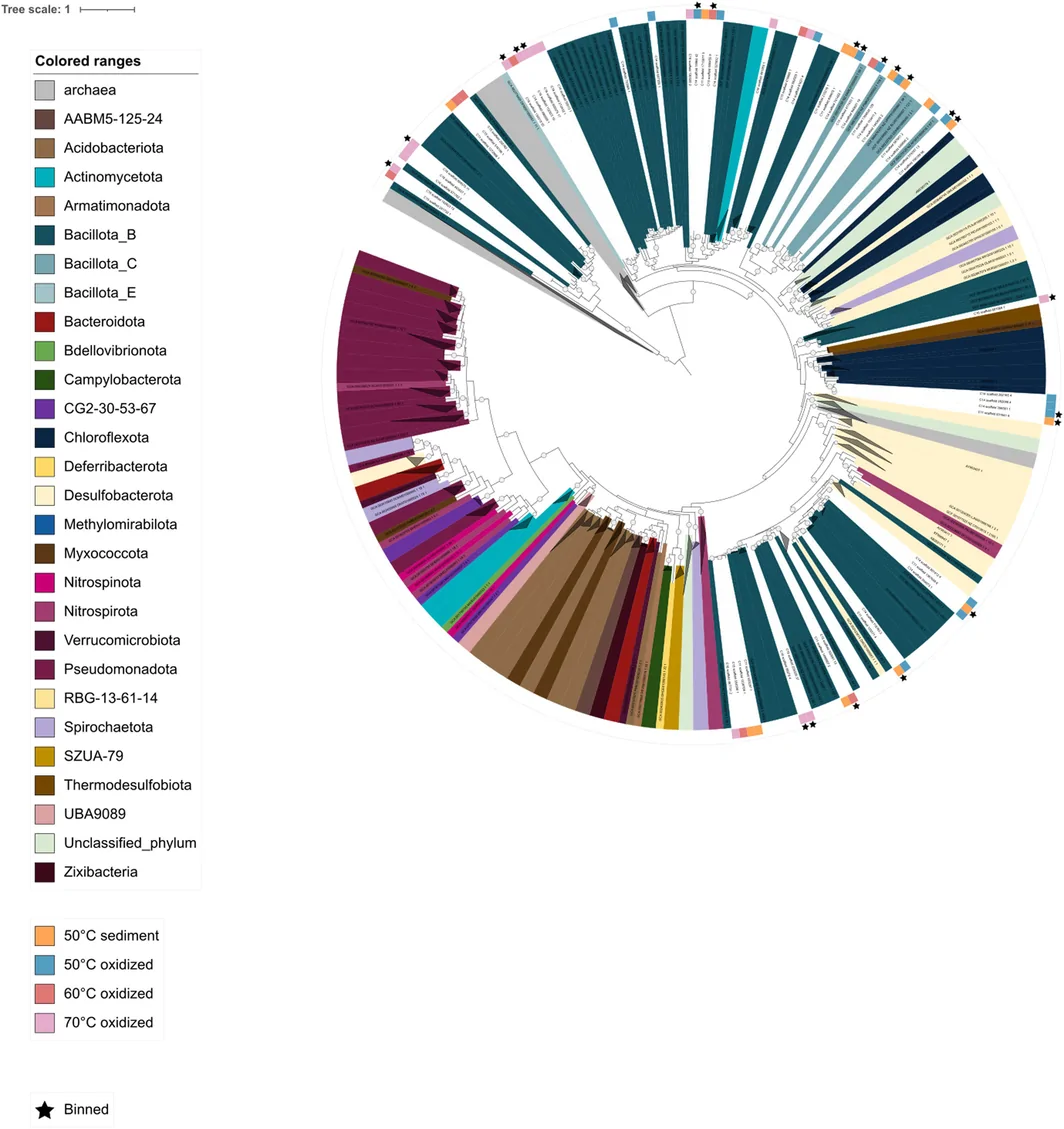
Phylogenetic Tree of dsrA proteins identified in the metagenomic assemblies from select temperatures and habitats. The dsrA tree includes fifty‐six dsrA proteins identified in the four metagenomic assemblies obtained in this study (Table S8), see the middle ring for proteins identified in this study and their associated samples. Twenty‐two of the dsrA proteins identified were found in MAGs, see the stars in the outer ring marking the binned protein sequences. The tree contains a reference dataset of dsrA sequences sourced from the public SILVA database (see methods). The phylogeny was inferred using IQ‐TREE v2.0.7, model LG + R10 (see methods).

Archaeal 16S rRNA sequencing detected archaea, particularly in 50°C sediment on week 16 (Table S3). These taxa (including *Methanosarcina*, *Methanocella*, *Methanobacterium*, and *Methanomassiliicoccus* spp.) are associated with methanogenesis, and metagenomic analysis supported these findings. Methanogenesis marker gene subunits, *mcrABG* and *mtrABCDE*, were annotated in medium and high‐quality MAGs from samples kept at 50°C (Figure 8). Genes associated with formate and methyl‐compound metabolism were also most represented in the 50°C samples in comparison to the higher temperatures (Figure 8). Genes associated with methanogenesis, as well as methane monooxygenase genes (*pMMO*) associated with methane oxidation, were found at all three temperatures, indicating metabolic potential for methane cycling across the temperature systems (Figure 8; Tables S4 and S6). Eukaryotic 18S sequencing yielded predominantly low‐abundance sequences and was therefore not considered informative for further analysis (NCBI SRA, accessions: SRR40259011, SRR40259013, SRR40259018; Table S7).

**FIGURE 8:**
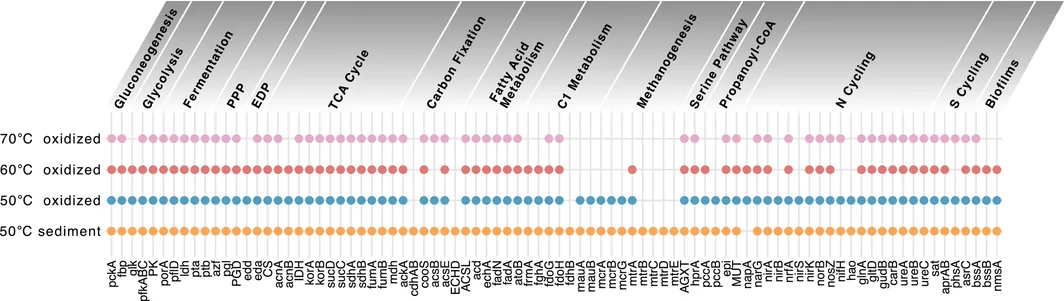
Presence of metabolic pathway genes across a sample of habitats in the impact hydrothermal system. Bubble plot diagram showing the presence and absence of genes related to various important metabolic pathways from quality checked MAGs (≥ 50% complete, < 10% contaminated) from select temperatures and habitats. PPP: Pentose phosphate pathway; EDP: Entner‐Doudoroff Pathway. Gene descriptions can be found in Table S8.

Genera associated with nitrogen cycling, including *Halomonas, Nitrospira, and Caldinitratiruptor* (Teske et al. 2000; Daims et al. 2015; Fardeau et al. 2010) were also detected. (Table S3). Metagenomic analysis further supported the presence of genetic potential for nitrogen cycling (Figure 8). Genes for nitrogenases used in nitrogen fixation (e.g., *nifH*, Figure 8) were identified, as were genes for nitrification (including ammonia monooxygenase; *pmoC‐amoC*) and nitrate reduction (e.g., *narG*, Figure 8).

The association of biofilms with oxidized suevite surfaces led us to hypothesize that these were sites of microbial iron‐cycling. Although we did identify sequences affiliated with iron‐oxidizing microorganisms, such as *Crenothrix*, *Leptothrix*, *Rhodoferax* spp. with presumptive iron‐oxidizing capabilities, they did not dominate the community. In support of the taxonomic data, our metagenomic analysis showed negligible abundance of iron oxidation genes (*Cyc2_repCluster2*, *Cyc2_repCluster3*), and limited presence of iron reduction genes (e.g., *Ndh2*, *PplA*, *FmnB*, *EetA*, *EetB*, *DmkB*, and *MtrA*). However, we observed an abundance of iron acquisition and regulation genes, especially for siderophore‐related transport and synthesis. (Tables S4 and S6). This suggests that while microbial communities in these regions have some potential for iron cycling, the biofilm might instead be associated with enhanced concentrations of iron availability as a nutrient rather than a redox component. Moreover, biofilm‐associated genes were identified across the metagenomic samples, with lower representation at 70°C (Figure 8).

Thermophiles from primarily heterotrophic lineages (e.g., *Meiothermus* and *Aneurinibacillus* spp.; Mori et al. 2012; Daims et al. 2015; Fardeau et al. 2010; Tsubouchi et al. 2015) were identified as dominant taxa across the sampled habitats (Figure 5). Metagenomic analysis identified genes associated with heterotrophic pathways (e.g., glycolysis, the Entner–Doudoroff pathway, the pentose phosphate pathway, and fermentation), which were strongly represented across habitats and temperatures (Figure 8).

## 4 Discussion

### 4.1 Rapid Geological Changes Establish Distinct Microbial Habitats

Owing to the lack of recently formed asteroid or comet impact craters on Earth today, knowledge of the rate at which microorganisms become established in the hydrothermal systems shortly after their formation is unavailable (Cockell and Lee 2002), in contrast to volcanic hydrothermal systems which can be microbiologically studied in the present day. The early events of microbial colonization of impact craters are therefore limited to inferences from the rock record (e.g., Drake et al. 2021; Hode et al. 2008; Ivarsson et al. 2013; Lindgren et al. 2010) or theoretical conjectures (Cockell and Lee 2002). To investigate microbial establishment and habitat development, we established artificial impact hydrothermal systems in the laboratory using post‐impact material from the Ries impact structure, Germany.

Geological changes were rapid. Within a week we observed oxidation of the hydrothermal substrate in the form of oxidation zones. Furthermore, the geological data showed that different habitats became established, including the surface oxidized suevite, the interior of the rocks beneath water level, and the formation of sedimentary habitats from the impact material, which disaggregated and formed a layer of sediment under the rocks or became dispersed within the fluid. The observation suggests that fluid–rock interactions rapidly establish distinct habitats for potential colonists. These habitats can only become available once the hydrothermal system has cooled to below the upper temperature limit for life (e.g., Kring et al. 2021), so that by the time microbial colonists can invade the structure, the substrate has likely already undergone geochemical alteration, including, for example, the partial or whole devitrification of glasses.

### 4.2 Rapid Enrichment of Diverse Microbial Communities in Impact Hydrothermal Substrates

The rate at which life can establish in impact hydrothermal systems after they have cooled to appropriate temperatures for life has been a matter of conjecture, although fossil record evidence suggests that colonization can occur soon after the formation of the system (Gustafsson et al. 2025).

Our data show empirically that microbial establishment and enrichment can be rapid. After 12 weeks we observed the formation of biofilms on the oxidized surfaces, the presence of endolithic communities within the suevite, and attachment to the surface sediment grains.

Furthermore, not only are distinct habitats formed rapidly after the impact event, but as soon as temperatures allow, each of these habitats can host varying microbial communities. Each of the habitats we observed shared some common taxa. This may reflect cosmopolitan physiological capabilities of the organisms and/or the dispersal of some cells throughout the hydrothermal system (therefore, reflecting the physical connection between the habitats rather than the specific capabilities of the taxa).

Especially noteworthy is that diverse thermophilic microbial communities were established in each of the three water baths, with a total of 340 genera detected across all microcosms. These observations are remarkable given that our inoculum was only 10 g of surface soil from a temperate biome. We chose this inoculum because impacts do not necessarily occur into existing volcanic high‐temperature hydrothermal systems with their associated biota. We chose to investigate what ecosystems would form from simulating wind‐blown inoculation of an impact hydrothermal system from nearby temperate soils. Whatever the controversies about Baas Becking's original conjecture that “everything is everywhere, but the environment selects” (Baas‐Becking 1934), this experiment is a demonstration that a large number of organisms are everywhere, and it is possible to assemble hyperthermophilic post‐impact communities from a few grams of temperate soils.

Variation in community composition among the different habitats may be linked to the geochemistry. For example, one hypothesis for the biofilm formation on oxidized suevite surfaces could be that the impact glasses provide preferential local regions of nutrient availability as these devitrify into clays (potentially smectites; Engelhardt et al. 1995). Basaltic glasses have been shown to weather faster than their mineral crystalline counterparts (Wolff‐Boenisch et al. 2006), and impact glasses may similarly provide improved sites of elemental leaching, including iron, that could support microbial growth. However, our results derive from a single crater material and an experimentally generated set of alteration habitats. The formation of biomass‐rich oxidized surfaces may therefore depend on substrate properties such as glass abundance, glass‐to‐clay ratios, alteration state, and fluid chemistry. These observations suggest one possible way in which impact‐derived substrates could influence microbial habitat development in post‐impact hydrothermal systems.

The high abundance of Firmicutes that we observed might be attributed to their spore‐forming ability, potentially facilitating survival across varying environmental challenges. Only *Thermaerobacter* was present in all temperatures and habitats. Similarly, most cultured organisms were Firmicutes (*Thermaerobacter*), which may reflect the presence of thermophilic spore‐formers in the temperate soil we used to inoculate the hydrothermal systems. Many of the thermophiles in this soil were possibly present as spores rather than actively metabolizing organisms, as spores of thermophiles have also been detected in temperate soils (Portillo et al. 2012).

Dominant taxa (Figure 5) include *Brockia*, for which the only cultured species, *Brockia lithotrophica*, uses molecular hydrogen or formate as electron donors, and elemental sulfur, thiosulfate or polysulfide as electron acceptors (Perevalova et al. 2013). *Caldinitratiruptor* was also abundant, and the only cultured species currently described from this genus, *Caldinitratiruptor microaerophilus*, is a microaerophilic nitrate‐reducing bacterium (Fardeau et al. 2010). Additionally, *Sphaerobacter* and *Thermomicrobium* dominated, which include organisms capable of using a range of organic substrates. Prior to the 12‐week perturbation, organic substrates in the microcosms may have been derived from residual organic material in the suevite and soil inocula, or from necromass produced within the systems. In Week 12, organic material was also introduced in the form of yeast extract, alongside a second soil inoculum. Following this combined perturbation, several communities shifted in composition and some lineages became enriched. The main implication of this phase is that microbial communities in impact hydrothermal microcosms can change following transient biological and organic inputs.

### 4.3 Post‐Impact Hydrothermal Systems Support Microbial Communities With Diverse Metabolic Potential

Metagenomic analysis of the communities showed that within 12 weeks diverse genes associated with cycling of key elements were present in the communities. These included genes linked to carbon cycling (including methane cycling), nitrogen cycling, and the transport and use of iron.

Sulfur cycling metabolisms are especially interesting in the context of post‐impact processes. Isotopic evidence for sulfate‐reduction has been reported in the rocks of several impact structures and has been attributed to post‐impact hydrothermal microbial activity (Kring et al. 2021; Parnell et al. 2010; Simpson et al. 2017). Our metagenomic annotations indicate that microorganisms encoding genes associated with sulfate reduction were present within 12 weeks in the impact hydrothermal microcosms. However, active sulfate reduction was not directly measured, and isotopic analysis of suevite at the end of the experiment did not yield bulk sulfur concentrations high enough to test for isotopic evidence of sulfate reduction. This suggests that such signatures may require longer periods of colonization, greater biomass accumulation, or more sustained activity to become concentrated enough to be preserved in the rock record. Nevertheless, our results indicate that, as impact hydrothermal environments cool to within the temperature range for life, microbial communities with the genetic potential for sulfate‐reduction, and its potential to leave an isotopic signature in the rock record, can become rapidly established in suevite‐hosted habitats. The metagenomic data show that a large diversity of sulfate‐reduction genes potentially capable of contributing to this process are represented (Figures 7 and 8).

Intriguingly, the carbon isotopic excursions associated with calcite in the fossil Lappajärvi impact hydrothermal system at formation temperatures lower than those associated with sulfate reduction led the authors to suggest that methane cycling occurred later in the sequence of hydrothermal events when the system had cooled (Gustafsson et al. 2025). In our experiment, methanogenesis‐associated genes and calcite rinds were identified. While these observations do not demonstrate methane cycling activity, they provide experimental context for the possibility that methanogenic potential may become established in lower‐temperature stages of impact hydrothermal systems.

### 4.4 Implications for Early Earth and Mars

Impact crater hydrothermal systems have been suggested to have provided habitats for life on early Earth, and this may have especially been the case during a period of heavy bombardment (Bottke et al. 2012; Cockell 2006; Osinski et al. 2020). Our experiment provides an analogue for examining how microbial communities from plausible modern source environments can become established in impact‐derived hydrothermal substrates over short timescales. In this system, taxonomically diverse and potentially functionally diverse thermophilic communities became established within distinctive habitats, including clay‐rich devitrified impact glass and the porous, fractured interiors of impact lithologies. These results indicate that impact‐derived hydrothermal substrates can support habitat differentiation and microbial enrichment over timescales of weeks to months.

Biogeochemical cycling is central to the habitability of hydrothermal systems, including impact‐generated environments. In our microcosms, organisms carrying genes associated with iron, sulfur, carbon, and nitrogen metabolism became established, indicating that impact‐derived hydrothermal substrates can support microbial communities with metabolic potential relevant to the cycling of key elements. The presence of methanogenic archaea and methanogenesis‐associated genes suggests that impact hydrothermal systems may host microbial communities with methanogenic potential, a process with possible implications for early atmospheric composition and planetary warming (Pavlov et al. 2000).

How these results translate to early Earth and Martian environments will depend on the microbial source pool, organic inventory, target lithology, fluid chemistry, atmospheric conditions, and the duration and thermal evolution of individual hydrothermal systems. Our experiment does not reproduce any single ancient planetary setting, because it used modern temperate soil inoculum, ancient altered suevite, modern oxygenated atmospheric conditions, and a controlled microcosm design. The oxygenated atmosphere likely influenced oxidative weathering of exposed suevite surfaces and may have favored aerobic or facultatively aerobic microorganisms that would be less directly applicable to low‐oxygen early Earth or early Mars environments. The oxidized surface habitats and associated biofilms should therefore be interpreted as products of the specific atmospheric and experimental conditions used here, rather than as direct analogues for all ancient impact hydrothermal settings. Even so, the experiment shows that impact‐derived hydrothermal substrates can support microbial establishment, habitat differentiation, and metabolic potential over short timescales, supporting their use as analogue environments for investigating processes relevant to early Earth and Martian exploration contexts.

## 5 Conclusion

This work demonstrates that impact‐derived hydrothermal substrates can support rapid microbial establishment, habitat differentiation, and diverse metabolic potential under laboratory conditions. These observations support the use of laboratory‐simulated impact crater hydrothermal systems as analogue environments for investigating the development of potentially habitable niches and biosignature formation in impact‐generated settings on planetary bodies.

Microbial biomass and biofilms developed in multiple habitats within the experimental systems, including oxidized suevite surfaces, endolithic suevite interiors, and sedimentary alteration products. These observations suggest that impact hydrothermal materials can generate a range of biomass‐bearing microhabitats with different preservation potential. Our data support the idea that impact‐generated hydrothermal systems can become sites of biomass accumulation and potential biosignature preservation over relatively short timescales (Drake et al. 2021; Glamoclija et al. 2007; Gustafsson et al. 2025; Hode et al. 2008; Ivarsson et al. 2013; Izawa et al. 2011; Lindgren et al. 2010). The association of biofilm‐rich oxidized suevite surfaces with carbonate formation indicates that some localities within impact hydrothermal systems may be promising targets for investigating ancient life in these systems. Within carbonates formed in the suevite, we observed many voids with microbial morphologies and sizes (Figure 5d), although the relationship between these textures and microbial activity needs to be investigated. Fourier Transform Infrared Spectroscopy (FTIR) also detected enhanced C‐H bands associated with the biofilm‐rich oxidized, demonstrating that organics associated with these biofilms can be detected spectroscopically, with potential implications for detecting remnant traces of life in planetary impact craters.

The rapid enrichment of microorganisms carrying genes associated with sulfate reduction is consistent with the idea that sulfur isotope systems may provide useful biosignature target in impact hydrothermal deposits, provided that appropriate geological, geochemical, and isotopic context is available (Kring et al. 2021; Parnell et al. 2010; Simpson et al. 2017; Tino et al. 2023). Although active sulfate reduction was not directly measured in our experiment, the presence of sulfate‐reduction‐associated genes within weeks to months indicates that impact‐derived hydrothermal substrates can support microbial communities with sulfur‐cycling potential over short timescales. The absence of sufficient sulfur for isotopic analysis, however, suggests that detectable geochemical traces of such activity may require longer timescales, greater biomass accumulation, or more sustained metabolic activity to be recorded in the rock substrate.

These findings support the use of impact hydrothermal systems as analogue environments for investigating processes relevant to early Earth and Martian exploration. Mars hosts abundant impact structures, many of which may have supported hydrothermal systems and fracture networks capable of providing habitats and preserving biosignatures (Newsom et al. 1986; Osinski et al. 2013; Robbins and Hynek 2012; Cockell et al. 2024). While our experiment does not directly reproduce early Mars or any single ancient planetary environment, it shows that impact‐derived hydrothermal substrates can support rapid microbial enrichment, habitat differentiation, biofilm formation, and metabolic potential. These processes strengthen the rationale for considering impact hydrothermal deposits as targets in the search for ancient or even extant life, and as environments of astrobiological interest in Martian exploration.

## Funding

This work was supported by Simons Foundation, 73592LPI, LI‐SIAME‐00002001. Science and Technology Facilities Council, ST/V000586/1, ST/Y001788/1. University of Texas Center for Planetary Systems Habitability (CPSH), #0093. University of Texas Institute for Geophysics (UTIG), #4195.

## Conflicts of Interest

The authors declare no conflicts of interest.

## Supporting information


**Figure S1:** Physical characteristics of the Reis impact suevite. (A) Plane polarized petrographic microscopy of raw collected suevite thin section. (B) Scanning Electron Microscope (SEM) backscattered electron image of raw collected suevite resin round showing large and irregular shaped vesicles. (C) BET (Brunauer–Emmett–Teller) analysis of suevite with BET summary values. (D) Pore size diameter distribution determined with mercury intrusion porosimetry. Highlighted green region represents pores accessible to microbes (> 1 μm diameter)—this is a maximum percentage estimate as information on pore throat diameters is not considered in the intrusion analysis.
**Figure S2:** Scanning Electron Microscopy of suevite material. (A) Stereoscope image of raw collected suevite resin round used for SEM‐EDS map. (B) SEM backscattered electron image of resin round with map boundaries drawn. Distribution of (C) Mg, Zn, Na and (D) Fe in raw collected suevite SEM‐EDS map.
**Figure S3:** Various identified minerals via SEM‐EDS in raw collected suevite. Bt = biotite; Ap = apatite; Qz = quartz; Zm = zircon; Sme = smectite; Cal = calcite; Pl = plagioclase; Kfs = K‐feldspar; G = glassy mesostasis; V = vesicle.
**Figure S4:** Harker diagram for various Fe wt% against Si wt% in raw collected suevite SEM‐EDS map. Iron content > 15 wt% is comparatively considered Fe‐rich.
**Figure S5:** Heat bath images showing visual changes in suevite from week 1 to 20. Notice the sedimentary layers forming over submerged rocks in 70°C, and significant characteristic oxidization on surfaces exposed above water level in 60°C and 70°C.
**Figure S6:** Time course of suevite oxidation in the hydrothermal system. Images showing characteristic oxidization on the surfaces exposed above water level of the same few rocks in the 70°C heat baths from week 1 to week 20. Arrows point to characteristic oxidation. All scale bars are 2 cm.
**Figure S7:** Secondary SEM images of suevite and alteration zones. (A) raw collected suevite surfaces; (B–D) fracturing of characteristic oxidized surface from 70°C heat bath. Notice the crust appears loosely connected and is thin (∼8.4 μm; D).
**Figure S8:** XRD diffractograms for raw collected suevite. (A) Raw spectrum showing glassy amorphous ‘hump’; (B) Baseline corrected spectrum showing mineral peaks.
**Figure S9:** Increasing alkalinity of fluids in experimental hydrothermal systems over time. Water pH was measured throughout the experiment in heat baths containing suevite at 50°C, 60°C, and 70°C, alongside a room‐temperature suevite + water control.
**Figure S10:** ICP‐MS analysis of elemental concentrations in solution. (A) Concentrations of elements (by atomic number) measured via ICP‐MS on week 12 for the hydrothermal fluids at 50°C, 60°C, and 70°C. (B) Zoom in of ICP‐MS data for the major elements in biological significance sodium to zinc.
**Figure S11:** 3D visualization of z‐stacks taken with confocal microscopy on oxidized suevite surfaces at 50°C, 60°C, and 70°C. Biofilm densities are written beneath the side (Z‐X plane) views.
**Figure S12:** Secondary SEM visualization of biofilms on oxidized of suevite. (A) zoomed out region showing fractured 70°C oxidized surface with arrows indicating location of cells; (B) zoomed in region of biofilm formation on 70°C oxidized suevite.
**Figure S13:** Secondary SEM images of 50°C of cells in submerged suevite. (A) in grain boundaries; (B) on surfaces; (C) in pores; (D) on surfaces, notice the arrow pointing to potentially encrusted microbes.
**Figure S14:** Stained cells attached to sediment grains. Fluorescence imaging of cells superimposed over brightfield images of grains in 50°C, 60°C, and 70°C sediment samples.
**Figure S15:** FTIR (Fourier Transform Infrared Spectroscopy) analysis of organics within the suevite. (a) Full FTIR spectra of altered suevite from various temperatures and habitats, with raw suevite as a comparison. (b) Zoom of yellow highlighted region in (a), corresponding to detection of organics (aliphatic C‐H stretching), most significantly in 60°C oxidized suevite; 60°C oxidized suevite peaks are 8.8× (for 2919 cm^−1^) and 6× (for 2851 cm^−1^) greater than the raw suevite sample at the same wavenumbers.
**Figure S16:** Dissimilarity analysis between experimental samples and inocula. (a) Genus‐level Bray–Curtis dissimilarity across all sampled habitats at weeks 9 and 16. (b) Genus‐level Bray–Curtis dissimilarity between experimental samples and the three inocula at weeks 9 and 16. Higher Bray–Curtis dissimilarity values indicate greater differences and therefore more distinct microbial communities. (c) ASV overlap between experimental samples and the three source communities (Soil 1, Soil 2, and suevite) at weeks 9 and 16. Shared‐source ASVs were those detected in more than one inoculum. ASV presence or absence in the inocula was used to assess overlap, but individual taxa cannot necessarily be assigned to a specific source unambiguously, as noted in the main text.
**Figure S17:** Distribution of bacterial genera across hydrothermal habitats and temperature systems. Venn diagrams based on bacterial 16S rRNA gene amplicon sequencing showing shared and unique genera across habitats and across the 50°C, 60°C, and 70°C systems on week 9 and 16.
**Figure S18:** Relative abundance of bacterial genera detected in the heat‐bath communities. Genera are based on bacterial 16S rRNA gene amplicon sequencing. Percentages are calculated relative to the pooled total read abundance across sampled temperatures and habitats.
**Figure S19:** Cell enumerations [cells/mL] in each of the heat baths (50°C, 60°C, and 70°C). Control count was taken in week 11, 6 days before the addition of organics and second soil inoculum which were added in week 12. All data series are plotted with (filled) error bars representing one standard deviation.

**Table S1:** XRD (X‐Ray Diffraction) analysis of suevite. Semi‐quantitative XRD raw and altered (weeks 8 and 17) suevite mineralogical concentrations in wt%. Data is normalized to a total 100 wt%.

**Table S2:** Isotope analysis of oxidized altered surface suevite. Samples taken on week 17. δ^13^C values are reported relative to V‐PDB scale using calibrated reference materials.

**Table S3:** Absolute abundances of genera assignments for bacterial and archaeal 16 s analysis.

**Table S4:** Phylogenetic affiliations of genome bins.

**Table S5:** Genes for sulfate reduction, nitrogen cycling and methanogenesis.

**Table S6:** FeGenie summary.

**Table S7:** Sequencing assays and NCBI SRA accession numbers.

**Table S8:** Specific pathway marker genes. Associated data for Figures 7 and 8: metabolic pathways and dsrA phylogeny. metabolic pathways: annotation guidelines for consideration of a gene hit from kofamscan. dsrA: all dsrA sequences from this studies samples included in the dsrA protein phylogeny. kofam: kofam annotations for high quality bins (≥ 90% complete, < 10% contaminated).

## Data Availability

Raw sequencing data generated in this study have been deposited in the NCBI Sequence Read Archive (SRA) under BioProject PRJNA1515288. Individual SRA accession numbers for samples are provided in Table S7. All other relevant extended data are provided in the Supporting Information.
