# Supplementary material for: Impact Crater Hydrothermal Systems Support Rapid Microbial Enrichment and Diverse Metabolic Potential
Source: Geobiology. 2026 Oct 17;24(6):e70070. doi: 10.1111/gbi.70070 (PMC13675768; doi:10.1111/gbi.70070)
Supplement: Supplementary file 1 [file GBI-24-e70070-s002.docx]

**Extended data**

Early impact crater hydrothermal systems are sites of high microbial diversity and biogeochemical cycles

**Extended Data Fig. 1. Physical characteristics of the Reis impact suevite.** (A) Plane polarised petrographic microscopy of raw collected suevite thin section. (B) Scanning Electron Microscope (SEM) backscattered electron image of raw collected suevite resin round showing large and irregular shaped vesicles. (C) BET (Brunauer-Emmett-Teller) analysis of suevite with BET summary values. (D) Pore size diameter distribution determined with mercury intrusion porosimetry. Highlighted green region represents pores accessible to microbes (>1μm diameter) - this is a maximum percentage estimate as information on pore throat diameters is not considered in the intrusion analysis.

**Extended Data Fig. 2. Scanning Electron Microscopy of suevite material.** (A) Stereoscope image of raw collected suevite resin round used for SEM-EDS map. (B) SEM backscattered electron image of resin round with map boundaries drawn. Distribution of (C) Mg, Zn, Na and (D) Fe in raw collected suevite SEM-EDS map.

**Extended Data Fig. 3. Various identified minerals via SEM-EDS in raw collected suevite**. Bt = biotite; Ap = apatite; Qz = quartz; Zm = zircon; Sme = smectite; Cal = calcite; Pl = plagioclase; Kfs = K-feldspar; G = glassy mesostasis; V = vesicle.

**Extended Data Fig. 4.** **Harker diagram for various Fe wt% against Si wt% in raw collected suevite SEM-EDS map**. Iron content >15 wt% is comparatively considered Fe-rich.

**Extended Data Fig. 5. Heat bath images showing visual changes in suevite from week 1 to 20.** Notice the sedimentary layers forming over submerged rocks in 70°C, and significant characteristic oxidization on surfaces exposed above water level in 60°C and 70°C.

**Extended Data Fig. 6. Time course of suevite oxidation in the hydrothermal system.** Images showing characteristic oxidization on the surfaces exposed above water level of the same few rocks in the 70°C heat baths from week 1 to week 20. Arrows point to characteristic oxidation. All scale bars are 2cm.

**Extended Data Fig. 7. Secondary SEM images of suevite and alteration zones.** (A) raw collected suevite surfaces; (B-D) fracturing of characteristic oxidized surface from 70◦C heat bath. Notice the crust appears loosely connected and is thin (∼8.4μm; D.)

**Extended Data Fig. 8. XRD diffractograms for raw collected suevite.** (A) Raw spectrum showing glassy amorphous ’hump’; (B) Baseline corrected spectrum showing mineral peaks.

**Extended Data Fig. 9. Increasing alkalinity of fluids in experimental hydrothermal systems over time**. Water pH was measured throughout the experiment in heat baths containing suevite at 50°C, 60°C, and 70°C, alongside a room-temperature suevite + water control.

**Extended Data Fig. 10. ICP-MS analysis of elemental concentrations in solution.** (A) Concentrations of elements (by atomic number) measured via ICP-MS on week 12 for the hydrothermal fluids at 50°C, 60°C, and 70°C. B) Zoom in of ICP-MS data for the major elements in biological significance sodium to zinc.

**Extended Data Fig. 11. 3D visualization of z-stacks taken with confocal microscopy on oxidized suevite surfaces at 50°C, 60°C, and 70°C.** Biofilm densities are written beneath the side (Z-X plane) views.

**Extended Data Fig. 12. Secondary SEM visualization of biofilms on oxidised of suevite**. (A) zoomed out region showing fractured 70°C oxidized surface with arrows indicating location of cells; (B) zoomed in region of biofilm formation on 70°C oxidized suevite.

**Extended Data Fig. 13. Secondary SEM images of 50°C of cells in submerged suevite**. (A) in grain boundaries; (B) on surfaces; (C) in pores; (D) on surfaces, notice the arrow pointing to potentially encrusted microbes.

**Extended Data Fig. 14. Stained cells attached to sediment grains.** Fluorescence imaging of cells superimposed over brightfield images of grains in 50°C, 60°C, and 70°C sediment samples.

**Extended Data Fig. 15. FTIR (Fourier Transform Infrared Spectroscopy) analysis of organics within the suevite.** (a) Full FTIR spectra of altered suevite from various temperatures and habitats, with raw suevite as a comparison. (b) Zoom of yellow highlighted region in (a), corresponding to detection of organics (aliphatic C-H stretching), most significantly in 60°C oxidized suevite; 60°C oxidized suevite peaks are 8.8x (for 2919 cm^-1^) and 6x (for 2851 cm^-1^) greater than the raw suevite sample at the same wavenumbers.

**Extended Data Fig. 16. Dissimilarity analysis between experimental samples and inocula.** (a) Genus-level Bray–Curtis dissimilarity across all sampled habitats at weeks 9 and 16. (b) Genus-level Bray–Curtis dissimilarity between experimental samples and the three inocula at weeks 9 and 16. Higher Bray–Curtis dissimilarity values indicate greater differences and therefore more distinct microbial communities. (c) ASV overlap between experimental samples and the three source communities (Soil 1, Soil 2, and suevite) at weeks 9 and 16. Shared-source ASVs were those detected in more than one inoculum. ASV presence or absence in the inocula was used to assess overlap, but individual taxa cannot necessarily be assigned to a specific source unambiguously, as noted in the main text.

**Extended Data Fig. 17. Distribution of bacterial genera across hydrothermal habitats and temperature systems.** Venn diagrams based on bacterial 16S rRNA gene amplicon sequencing showing shared and unique genera across habitats and across the 50, 60, and 70°C systems on week 9 and 16.

**Extended Data Fig. 18. Relative abundance of bacterial genera detected in the heat-bath communities.** Genera are based on bacterial 16S rRNA gene amplicon sequencing. Percentages are calculated relative to the pooled total read abundance across sampled temperatures and habitats.

**Extended Data Fig. 19. Cell enumerations [cells/mL] in each of the heat baths (50°C, 60°C, and 70°C.)** Control count was taken in week 11, 6 days before the addition of organics and second soil inoculum which were added in week 12. All data series are plotted with (filled) error bars representing one standard deviation.
